# The gut microbiome in polycystic ovary syndrome: mechanistic pathways and therapeutic potential of microbiota-targeted interventions

**DOI:** 10.3389/fendo.2026.1900535

**Published:** 2026-08-21

**Authors:** Farah Elhennawy, Basmalah Naji, Alexandra E. Butler

**Affiliations:** 1 School of Medicine, Royal College of Surgeons in Ireland-Medical University of Bahrain, Busaiteen, Bahrain; 2 Research Department, Royal College of Surgeons in Ireland-Medical University of Bahrain, Busaiteen, Bahrain

**Keywords:** dietary interventions, gut dysbiosis, gut microbiome, insulin resistance, polycystic ovary syndrome, probiotics, synbiotics

## Abstract

Polycystic Ovary Syndrome (PCOS) is one of the most common endocrine disorders worldwide, affecting 6–13% of reproductive-aged women and exerting a profound metabolic, reproductive and psychological toll. Emerging evidence suggests that the gut microbiome may be a potentially critical, yet under-recognized, contributor to the pathophysiology of PCOS. Alterations in microbial composition and function appear to influence hormonal imbalance, metabolic dysfunction and inflammatory processes that characterize the condition. However, much of the current evidence remains associative or derived from preclinical models, and the causal nature of these relationships is only beginning to be established through approaches such as Mendelian randomization. This literature review examines the evolving relationship between the gut microbiome and PCOS through a mechanism-guided, evidence-stratified framework, with particular focus on how microbiota-targeted interventions — including prebiotics, probiotics, synbiotics, dietary modifications and fecal microbiota transplantation, may modulate gut health and mitigate symptom severity. Across the reviewed studies, microbiome-targeted supplementation was associated with improvements in insulin sensitivity, reductions in systemic inflammation and favorable hormonal changes, although the strength of evidence varies across outcomes and intervention types. Importantly, PCOS heterogeneity, including differences in body mass index (BMI), insulin resistance status and phenotype, may influence both gut microbiota composition and therapeutic response, underscoring the need for phenotype-stratified research. These findings suggest that targeting the gut microbiome may serve as a promising adjunct to conventional PCOS management. Though further rigorous, long-term clinical trials are needed to advance both understanding and clinical translation.

## 1 Introduction

Polycystic Ovary Syndrome (PCOS), recently proposed to be renamed polyendocrine metabolic ovarian syndrome (PMOS) (1), is a prevalent endocrine disorder affecting 6–13% of women of reproductive age (2). It is characterized by hyperandrogenism, ovulatory dysfunction, and polycystic ovarian morphology, leading to significant physical and psychological impacts (3). PCOS is a highly heterogeneous condition; according to the Rotterdam criteria, four distinct phenotypes (A–D) are recognized, with phenotype A being the most severe. They differ in their metabolic risk profiles, hormonal characteristics and clinical presentations (10). This heterogeneity extends to body composition, with approximately 50–70% of women with PCOS being overweight or obese, while a substantial proportion present with a lean phenotype that nonetheless exhibits metabolic and reproductive dysfunction (4).

Current management of PCOS is primarily directed toward symptom control and includes lifestyle modification and pharmacological therapy tailored to individual clinical concerns such as menstrual irregularity, infertility, hyperandrogenism and metabolic dysfunction. Lifestyle interventions focusing on weight reduction through dietary modification and physical activity are recommended as first-line therapy and have been shown to improve insulin sensitivity, ovulatory function, and treatment responsiveness (5). Pharmacologic options include combined oral contraceptives and progestin therapy for menstrual regulation and hyperandrogenic symptoms, ovulation-induction agents, insulin-sensitizing agents including metformin for metabolic abnormalities, and anti-androgens such as spironolactone for hirsutism and acne (5). Despite their widespread use, these approaches largely address downstream manifestations rather than the underlying pathophysiology of PCOS, and their efficacy may be limited by adverse effects, contraindications or suboptimal patient adherence (6). This has prompted increasing interest in novel adjunctive strategies targeting upstream mechanisms, including modulation of the gut microbiome (6).

Emerging evidence suggests the gut microbiome plays a role in PCOS pathogenesis, with observational studies demonstrating associations between gut dysbiosis and hormonal imbalance, inflammation and metabolic dysfunction in women with PCOS (7). However, it is important to note that much of this evidence remains correlative, derived from cross-sectional human studies or preclinical animal models, and the causal directionality of these associations is not yet fully established. Recent Mendelian randomization (MR) studies have begun to address this gap: Min et al. (8) conducted a bidirectional two-sample MR analysis and meta-analysis identifying six causal associations between specific gut microbial genera and PCOS risk, providing genetic evidence that certain microbial taxa may have a causal role rather than being purely secondary to the disease state (8). Additional MR studies have corroborated these findings, identifying both protective taxa (e.g., Sellimonas, Bilophila, Holdemania) and risk-enhancing taxa, with some analyses demonstrating that the causal effect of Sellimonas on PCOS is partially mediated through BMI (9, 10). Importantly, these MR studies also suggest that microbiome alterations may be both a cause and a consequence of PCOS, supporting the concept of bidirectional causal pathways, although causal inference remains limited by methodological constraints including the use of genus-level genetic instruments and potential pleiotropy (8, 10).

There is also growing interest in dietary interventions such as the ketogenic diet, the Mediterranean diet and DASH (Dietary Approach to Stop Hypertension), which have shown positive effects on weight loss, insulin sensitivity and reproductive function (11, 12). Prebiotics, probiotics and synbiotics, which modulate gut microbiota composition and function, show promise as novel approaches to PCOS symptom management (13).

Several recent reviews have addressed aspects of the gut microbiota–PCOS relationship, including systematic reviews of microbial composition and metabolic alterations (14), meta-analyses of probiotic/synbiotic interventions (15–18), and narrative reviews of mechanistic pathways (19). While these contributions have advanced the field, a gap remains in integrating mechanistic evidence with clinical intervention data through an evidence-stratified framework that accounts for PCOS heterogeneity and translational limitations. This literature review aims to address this gap by: (1) systematically stratifying evidence by level (observational associations, mechanistic hypotheses from preclinical models, and causal inference from MR studies); (2) examining how PCOS phenotype, BMI and insulin resistance status may influence gut microbiota composition and therapeutic response; (3) linking specific dysregulated pathways to targeted interventions; and (4) critically appraising the clinical evidence base for microbiota-targeted therapies, including their limitations and translational barriers.

## 2 Methods

A literature review was conducted on established databases, including PubMed, Google Scholar, and Scopus, to ensure comprehensive coverage of the relevant literature. A well-defined search strategy was implemented using keywords “Polycystic Ovary Syndrome,” “PCOS,” “gut microbiome,” “gut dysbiosis,” “prebiotics,” “symptoms,” and “dietary interventions.” The full search strategy for each database is provided in **Supplementary Table 1**. Inclusion criteria included peer-reviewed articles published in English, studies focusing on the gut microbiome’s impact on PCOS, those discussing dietary interventions, and articles published in the past 10 years (2016–2026) to ensure inclusion of the most recent and methodologically robust evidence, particularly given the rapidly evolving nature of gut microbiome research and advancements in sequencing technologies that have significantly improved the understanding of microbiota-related mechanisms in PCOS. Exclusion criteria include articles that do not focus on women of reproductive age, studies not available in English, research not directly related to the gut microbiome or dietary interventions, and those published more than 10 years ago. As this is a literature review, formal systematic screening procedures, data extraction protocols, quality assessment or risk-of-bias evaluation were not performed.

## 3 PCOS pathophysiology and its relationship with metabolism, inflammation and the gut microbiome

### 3.1 Pathophysiology of PCOS

PCOS is a multifaceted endocrine disorder characterized by a constellation of hormonal, metabolic and reproductive abnormalities. According to the Rotterdam criteria, diagnosis requires the presence of at least two of the following three features: oligo- or anovulation, clinical or biochemical hyperandrogenism, and polycystic ovarian morphology on ultrasound. This had led to the recognition of distinct PCOS phenotypes, including phenotype A (classic PCOS with all three features), phenotype B (hyperandrogenism and anovulation), phenotype C (hyperandrogenism with ovulatory cycles), and phenotype D (anovulation with polycystic ovarian morphology but no hyperandrogenism) (20). The different phenotypes of PCOS are presented in **Table 1**.

**Table 1:** Diagnostic features for different PCOS phenotypes (A-D).

Phenotype	Diagnostic features (Rotterdam criteria)	Clinical notes
Phenotype A (Classic PCOS)	Hyperandrogenism + oligo/anovulation + polycystic ovaries	Most severe phenotype; highest metabolic risk
Phenotype B	Hyperandrogenism + oligo/anovulation	Similar to classic PCOS but without ultrasound PCO morphology
Phenotype C (Ovulatory PCOS)	Hyperandrogenism + polycystic ovaries	Often milder reproductive dysfunction; metabolic risk still present
Phenotype D (Non-hyperandrogenic PCOS)	Oligo/anovulation + polycystic ovaries	Least “androgenic” phenotype; different hormonal profile

Key hormonal features include elevated levels of androgens, such as testosterone, and imbalances in luteinizing hormone (LH) and follicle-stimulating hormone (FSH) (21). In women with PCOS, raised levels of LH cause excess production of ovarian thecal androgens, whereas relative deficiency of FSH causes follicular arrest, polycystic ovarian morphology and oligo-ovulation (22). This hormonal imbalance often leads to impaired ovarian function, resulting in irregular menstrual cycles and anovulation, contributing to infertility in affected women (21, 22).

Metabolically, women with PCOS frequently exhibit insulin resistance, where cells fail to respond effectively to insulin. This resistance can lead to compensatory hyperinsulinemia, worsening symptoms such as weight gain, and increasing the risk of developing type 2 diabetes (4). Insulin resistance is defined as a pathological condition characterized by decreased responsiveness or sensitivity to the metabolic actions of insulin and plays an influential role in the development and persistence of PCOS. Additionally, obesity is prevalent among PCOS patients, further complicating the clinical picture. Obesity further complicates PCOS by exacerbating insulin resistance and contributing to inflammatory states (4). However, it is important to note that insulin resistance and metabolic dysfunction also occur in lean women with PCOS, suggesting that mechanisms beyond adiposity contribute to the metabolic phenotype (4, 23).

Common comorbidities associated with PCOS include metabolic syndrome, which encompasses a cluster of conditions such as hypertension, dyslipidemia and central obesity, that collectively increase the risk of cardiovascular disease (4). The hormonal and metabolic dysregulation in PCOS often leads to chronic low-grade inflammation, which can further disrupt metabolic processes and exacerbate symptoms (4).

### 3.2 The gut microbiome in PCOS pathogenesis: an overview

**Figure 1:**
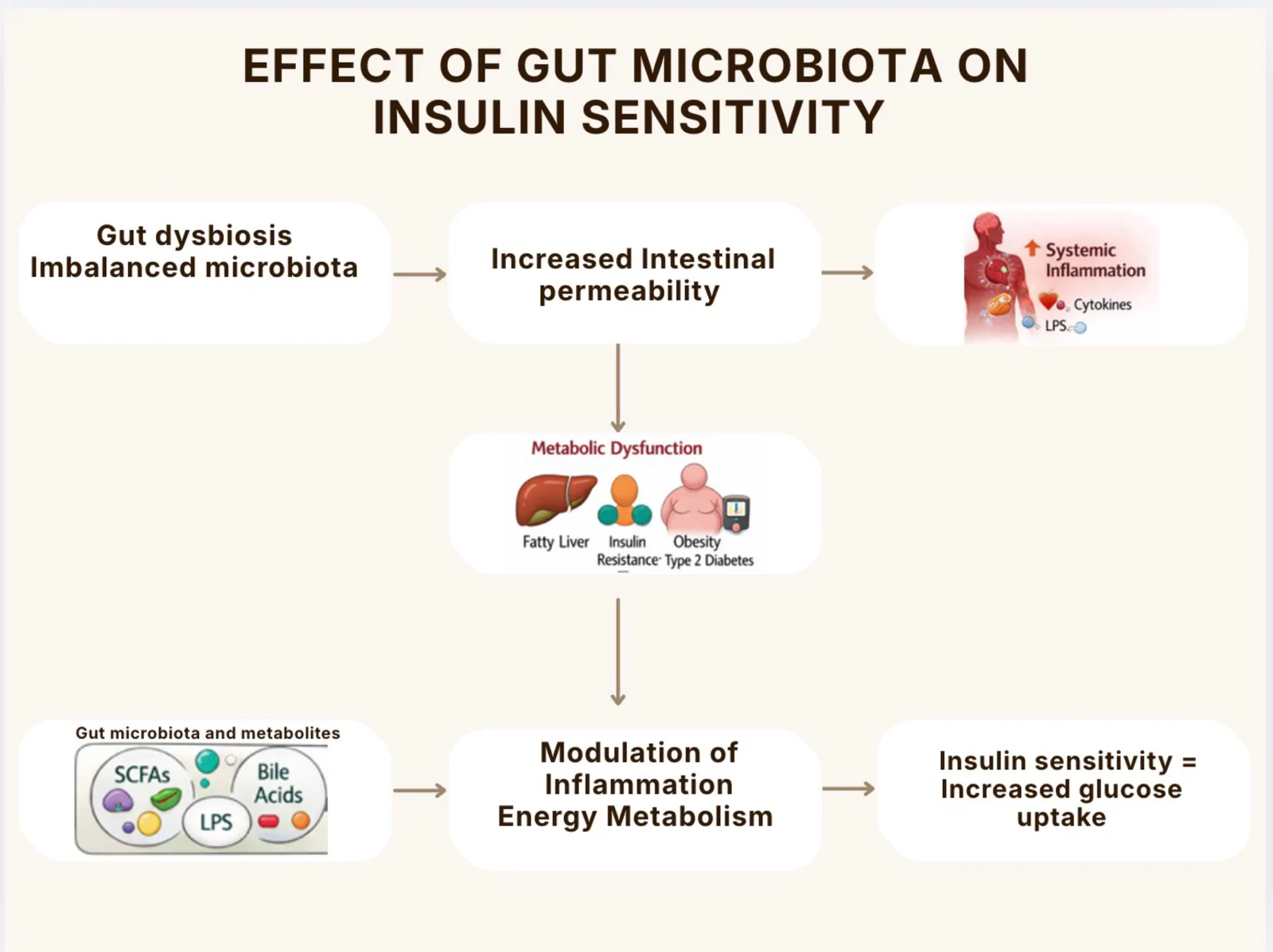
Flow chart diagram showing the effect of gut microbiota on insulin sensitivity. Figure created using Canva (Canva Pty Ltd).

Emerging research suggests that gut dysbiosis, or imbalances in gut microbiota composition, may play a significant role in the pathogenesis of PCOS. Gut dysbiosis is associated with increased intestinal permeability, leading to heightened systemic inflammation and metabolic dysfunction (7). The gut microbiota influences insulin sensitivity through the production of metabolites that can modulate inflammation and energy metabolism, thereby affecting the severity of PCOS symptoms. This is illustrated in **Figure 1** (7).

## 4 Gut dysbiosis in PCOS: microbial alterations and heterogeneity across studies

### 4.1 Microbial composition changes

Studies employing 16S rRNA and shotgun metagenomic sequencing have revealed alterations in the gut microbiota of women with PCOS compared to healthy controls. Several studies have reported reduced alpha diversity and shifts in beta diversity in PCOS patients, although these findings are not universal (14, 24). At the phylum level, an elevated ratio of *Firmicutes* to *Bacteroidetes* has been observed in some PCOS cohorts and has been associated with obesity and metabolic syndrome (14). Increased *Proteobacteria* levels have been linked to inflammation and metabolic dysregulation (14).

At the genus and species level, findings are more heterogeneous. Some studies report decreased abundance of *Prevotella* and increased *Bacteroides* in PCOS patients with insulin resistance (14), while others, notably the landmark study by Qi et al. (25), demonstrated that *Bacteroides vulgatus* was markedly elevated in women with PCOS and contributed to disease pathogenesis through disruption of bile acid metabolism (25). This apparent inconsistency highlights that the role of specific taxa may be species-specific and context-dependent, and that entire genera should not be simplistically classified as “beneficial” or “harmful.” Reductions in short-chain fatty acid (SCFA)-producing genera such as *Bifidobacterium, Roseburia* and *Lachnospira* have been more consistently reported across studies (7, 14).

### 4.2 Influence of PCOS heterogeneity on gut microbiota composition

A critical consideration in interpreting gut microbiome studies in PCOS is the substantial heterogeneity of the condition itself. BMI, insulin resistance status, PCOS phenotype, diet, medication use (including metformin and oral contraceptives), ethnicity and sequencing methodology may all influence gut microbiota composition and contribute to inconsistent findings across studies (23, 24).

BMI-stratified analyses have revealed important differences. Su et al. (2025) systematically reviewed BMI-stratified PCOS phenotypes and found that gut dysbiosis in the obese subgroup is generally more pronounced, potentially amplifying metabolic abnormalities through SCFA deficiency, bile acid disturbances and endotoxin-related low-grade inflammation (23). In contrast, non-obese PCOS primarily manifests as reproductive endocrine dysfunction, and the gut microbiota alterations in this subgroup may be qualitatively different, with less pronounced metabolic dysbiosis but potentially distinct hormonal and immune-mediated pathways (23). Insenser et al. (24) demonstrated that beta diversity was reduced particularly in obese patients with PCOS, and that bacterial diversity correlated positively with serum androgen concentrations and negatively with estradiol levels, suggesting that sex hormones themselves may shape the gut microbiome (24). Jobira et al. (26) found that even when comparing obese adolescents with and without PCOS (matched for BMI), those with PCOS had decreased alpha diversity and distinct taxonomic profiles, with lower *Bacteroidaceae* conferring a 4.4-fold likelihood ratio for PCOS, suggesting that PCOS-specific microbial alterations exist independently of obesity (26). Li et al. (27) further demonstrated that PCOS patients with BMI ≥ 24 had multiple dominant genera distinct from those with BMI < 24, confirming that body composition modulates the PCOS-associated microbiome (27).

These findings underscore the importance of phenotype-stratified analyses in future microbiome studies and suggest that a “one-size-fits-all” characterization of the PCOS gut microbiome is insufficient. Future studies should systematically stratify participants by PCOS phenotype (A–D), BMI, insulin resistance status and medication use to enable more precise characterization of disease-relevant microbial signature.

## 5 Mechanisms linking the gut microbiome to PCOS pathophysiology

**Figure 2:**
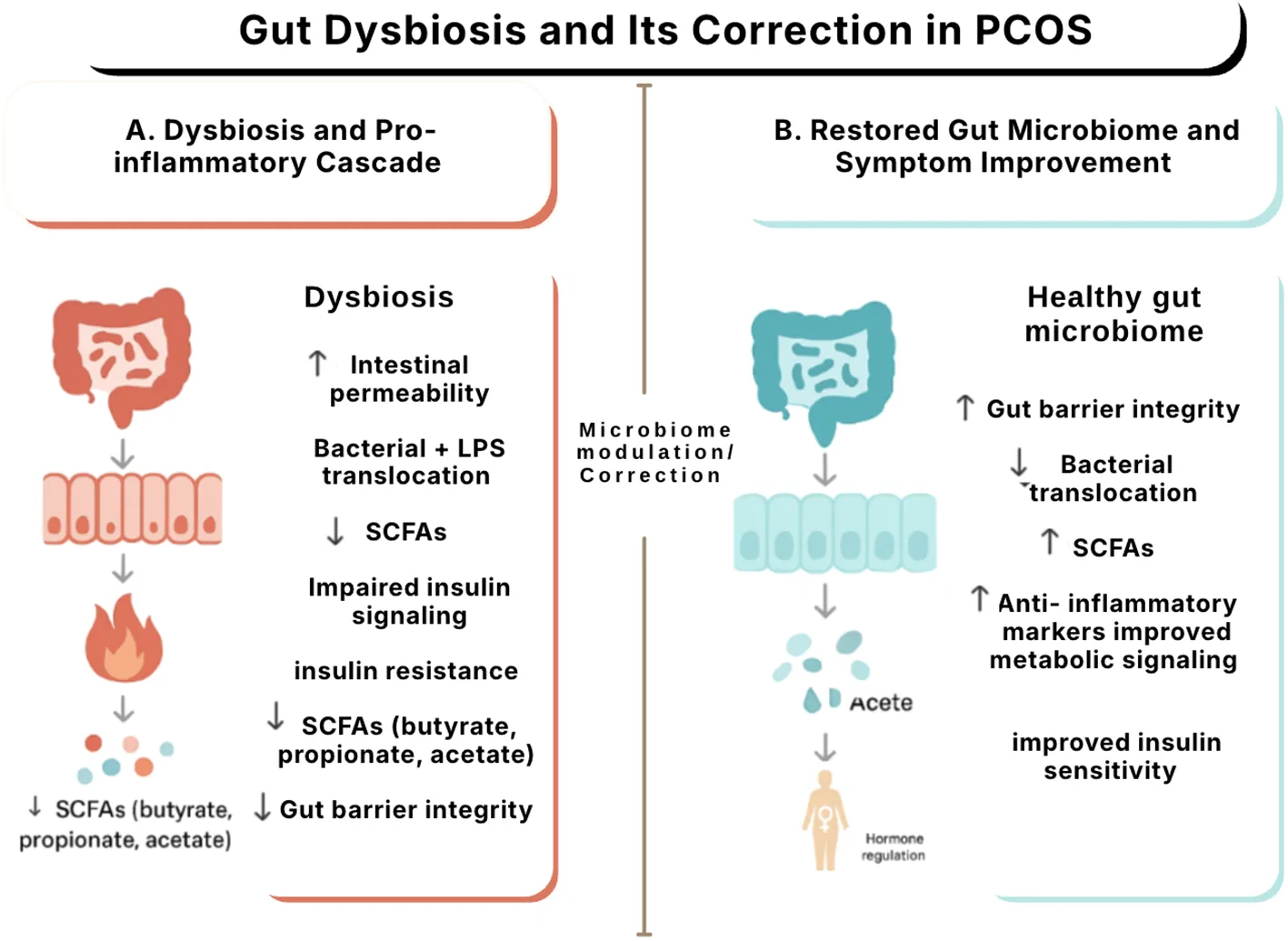
Gut dysbiosis and its correction in polycystic ovary syndrome (PCOS). **(A)** Dysbiosis and pro-inflammatory cascade, illustrating gut dysbiosis and its downstream effects, including increased intestinal permeability, bacterial and LPS translocation, reduced SCFAs, impaired insulin signaling, insulin resistance, and reduced gut barrier integrity. **(B)** Restored gut microbiome and symptom improvement following microbiome-modulating interventions, showing improved gut barrier integrity, reduced bacterial translocation, increased SCFAs, enhanced anti-inflammatory markers, improved metabolic signaling, and improved insulin sensitivity. Figure created using Canva (Canva Pty Ltd).

The gut microbiome influences PCOS pathophysiology through several interconnected molecular pathways. Importantly, many of these pathways are also implicated in obesity and type 2 diabetes (T2D), conditions that frequently co-occur with PCOS. Given the high prevalence of obesity and insulin resistance in PCOS (4), understanding these shared mechanisms provides useful context, although evidence derived from obesity and T2D cannot be directly equated with PCOS-specific pathophysiology. Where possible, the following sections distinguish between evidence obtained directly in PCOS populations, evidence extrapolated from related metabolic conditions, and mechanistic hypotheses requiring further validation. This interplay is illustrated in **Figure 2**.

### 5.1 Short-chain fatty acids and metabolic regulation

Short-chain fatty acids (SCFAs), principally acetate, propionate and butyrate, are produced by microbial fermentation of dietary fiber and represent a primary mechanism through which the gut microbiota influences host metabolism. SCFAs bind to G-protein-coupled receptors GPR41 (FFAR3) and GPR43 (FFAR2) expressed on enteroendocrine L-cells, adipocytes and immune cells, thereby regulating multiple metabolic processes (28). Activation of these receptors on L-cells stimulates the secretion of GLP-1 and peptide YY (PYY), hormones that enhance insulin secretion, improve insulin sensitivity and promote satiety (28, 29). Butyrate additionally serves as the primary energy source for colonocytes and maintains gut barrier integrity by upregulating tight junction proteins, thereby preventing the translocation of pro-inflammatory bacterial products (29).

In women with PCOS, observational studies have demonstrated reduced abundance of SCFA-producing bacteria such as *Bacteroides*, *Bifidobacterium* and *Roseburia*, which is associated with diminished SCFA production, impaired GLP-1 secretion, worsened insulin resistance and compromised gut barrier function (7, 30). However, it should be noted that these findings are largely derived from cross-sectional microbiome profiling studies and do not establish whether SCFA deficiency is a cause or consequence of PCOS-related metabolic dysfunction.

Supportive context from related conditions strengthens the biological plausibility of these observations. In obesity and T2D, reductions in SCFA-producing taxa have been consistently associated with increased energy harvest from the diet, enhanced adipogenesis and impaired glucose homeostasis (31–33). While this mechanistic overlap suggests that SCFA deficiency may represent a shared pathogenic node, direct interventional studies demonstrating that restoring SCFA production improves PCOS-specific outcomes are still needed.

### 5.2 Gut barrier dysfunction, endotoxemia and immune-inflammatory cascades

Gut dysbiosis in PCOS is associated with increased intestinal permeability, commonly referred to as “leaky gut,” which facilitates the translocation of lipopolysaccharides (LPS) from gram-negative bacteria into the systemic circulation, a state termed metabolic endotoxemia (33). Human observational studies in women with PCOS have demonstrated that serum levels of LPS, LPS-binding protein (LBP) and the LPS/HDL ratio are significantly elevated compared to healthy controls, and these markers correlate positively with testosterone levels, ovarian volume and hirsutism scores (33).

At the molecular level, LPS binds to Toll-like receptor 4 (TLR4) on immune cells, activating the nuclear factor-κB (NF-κB) and c-Jun N-terminal kinase (JNK) signaling cascades, which trigger the release of pro-inflammatory cytokines including tumor necrosis factor-alpha (TNF-α), interleukin-6 (IL-6) and interleukin-1 beta (IL-1β) (19, 34). This chronic low-grade inflammation directly impairs insulin receptor signaling through serine phosphorylation of insulin receptor substrate-1 (IRS-1), thereby exacerbating insulin resistance (34). Importantly, *in vitro* evidence has shown that LPS can directly stimulate androgen production in ovarian theca-interstitial cell cultures, providing a mechanistic link between endotoxemia and hyperandrogenism, although this finding awaits confirmation in human *in vivo* studies (33).

Beyond the LPS-TLR4 axis, dysbiotic conditions may also promote macrophage pyroptosis and the release of damage-associated molecular patterns (DAMPs), which amplify the inflammatory cascade (19). The IKK-NF-κB and JNK pathways, activated by both microbial products and inflammatory cytokines, may create a self-reinforcing cycle of inflammation that impairs insulin signaling, promotes androgen excess and disrupts ovarian folliculogenesis (19, 34). However, much of this evidence is derived from *in vitro* and animal studies, and the extent to which these amplification loops operate in women with PCOS remains to be fully characterized.

The LPS-TLR4-NF-κB pathway is also a well-established driver of insulin resistance in obesity and T2D, where it contributes to adipose tissue inflammation, hepatic steatosis and systemic metabolic dysfunction (31, 35). The convergence of this pathway across PCOS, obesity and T2D supports the concept of shared inflammatory mechanisms, while also highlighting that the PCOS-specific contribution of endotoxemia, particularly its direct effect on ovarian androgen production, may represent a distinct pathogenic feature.

### 5.3 Bile acid metabolism and nuclear receptor signaling

Bile acids, synthesized from cholesterol in the liver and extensively modified by gut bacteria through deconjugation, dehydroxylation and epimerization, function as potent signaling molecules that regulate glucose and lipid metabolism through the nuclear farnesoid X receptor (FXR) and the membrane-bound Takeda G-protein-coupled receptor 5 (TGR5) (36). Activation of intestinal FXR induces fibroblast growth factor 19 (FGF19), which suppresses hepatic bile acid synthesis and improves insulin sensitivity, while TGR5 activation on enteroendocrine L-cells stimulates GLP-1 secretion, further enhancing glucose homeostasis (36, 37).

Among the mechanistic pathways discussed in this review, the bile acid-FXR-IL-22 axis has the most direct PCOS-specific evidence. Qi et al. (25) demonstrated that *Bacteroides vulgatus*, which is markedly elevated in the gut microbiota of women with PCOS, disrupts bile acid metabolism by reducing levels of glycodeoxycholic acid (GDCA) and tauroursodeoxycholic acid (TUDCA) (25). Reduced TUDCA diminishes intestinal group 3 innate lymphoid cell (ILC3) secretion of interleukin-22 (IL-22) through impaired GATA3 signaling (38). IL-22 is critical for maintaining gut barrier integrity, regulating insulin sensitivity and modulating ovarian function; its deficiency in PCOS contributes to both metabolic and reproductive dysfunction (25).

More recently, Zhao et al. (39) extended these findings in a murine model, demonstrating that sialic acid (Neu5Ac) promotes the expansion of *Ligilactobacillus* salivarius, a bacterium with bile salt hydrolase activity that reduces TUDCA levels (39). The resulting enhancement of intestinal FXR activation suppresses IL-22 production and impairs STAT3 signaling, ultimately leading to increased ovarian ferroptosis and exacerbation of PCOS phenotypes (39). Together, these studies reveal a bile acid-FXR-IL-22 signaling axis as a specific and experimentally validated mechanistic pathway in PCOS pathogenesis, although it should be noted that the Zhao et al. findings are derived from animal models and require clinical confirmation.

The broader bile acid-FXR/TGR5 signaling axis is also central to the pathophysiology of obesity and T2D, where disrupted bile acid profiles contribute to impaired glucose tolerance, dyslipidemia, and hepatic steatosis (36, 37). The shared involvement of bile acid dysregulation across these conditions further supports the concept of common gut microbiome-mediated pathogenic mechanisms.

### 5.4 Hormonal modulation via the estrobolome

The gut microbiota may influence circulating estrogen levels through the “estrobolome,” the collection of bacterial genes encoding β-glucuronidase enzymes that deconjugate estrogens in the gut, allowing their reabsorption into the systemic circulation (30). Dysbiosis-associated reductions in β-glucuronidase activity could theoretically lead to decreased circulating estrogen levels, which may contribute to the relative estrogen deficiency and anovulation observed in PCOS (30, 40).

Additionally, gut microbiota-derived metabolites may influence the hypothalamic-pituitary-gonadal (HPG) axis through the gut-brain axis, potentially affecting gonadotropin-releasing hormone (GnRH) pulsatility and the LH/FSH ratio that drives ovarian androgen production (30, 40).

However, it should be acknowledged that direct evidence linking estrobolome dysfunction specifically to PCOS pathophysiology remains preliminary. Most evidence for the estrobolome concept is derived from studies in postmenopausal women and breast cancer populations, and its relevance to the hormonal milieu of reproductive-age women with PCOS has not been systematically investigated. This pathway should therefore be considered a plausible but unvalidated hypothesis requiring dedicated study in PCOS cohorts.

Importantly, these mechanistic pathways do not operate in isolation. LPS-mediated activation of the TLR4-NF-κB cascade damages the intestinal epithelium, reducing the colonic environment favorable to SCFA-producing bacteria and thereby diminishing butyrate availability — creating a vicious cycle of barrier loss, inflammation and SCFA depletion (29, 33, 34). Concurrently, inflammation-driven shifts in microbial composition can alter bile acid-metabolizing bacteria, disrupting the bile acid–FXR–IL-22 axis and compounding both metabolic and reproductive dysfunction (25, 36). The resulting IL-22 deficiency further impairs gut barrier maintenance, linking bile acid dysregulation back to endotoxemia. This interconnected nature of the pathways suggests that interventions targeting multiple nodes simultaneously may offer greater therapeutic potential than single-mechanism approaches, although the dynamics of these inter-pathway interactions remain poorly characterized in PCOS populations.

## 6 Microbiota-targeted interventions and dietary approaches in PCOS

In addition to direct microbiota supplementation, broader dietary patterns have been studied for their impact on PCOS through gut microbiome modulation. Low glycemic index (GI) diets, which focus on whole grains, legumes and fiber-rich foods, help stabilize blood glucose levels and may improve insulin sensitivity and ovulatory function. Mediterranean diets, rich in omega-3 fatty acids, fiber and anti-inflammatory foods such as fruits, vegetables, healthy fats and lean proteins, have been associated with improvements in menstrual regularity, insulin resistance and cardiovascular health. These dietary patterns may exert their beneficial effects in part through favorable modifications of gut microbiota composition and enhanced SCFA production (12, 43).

**Figure 3:**
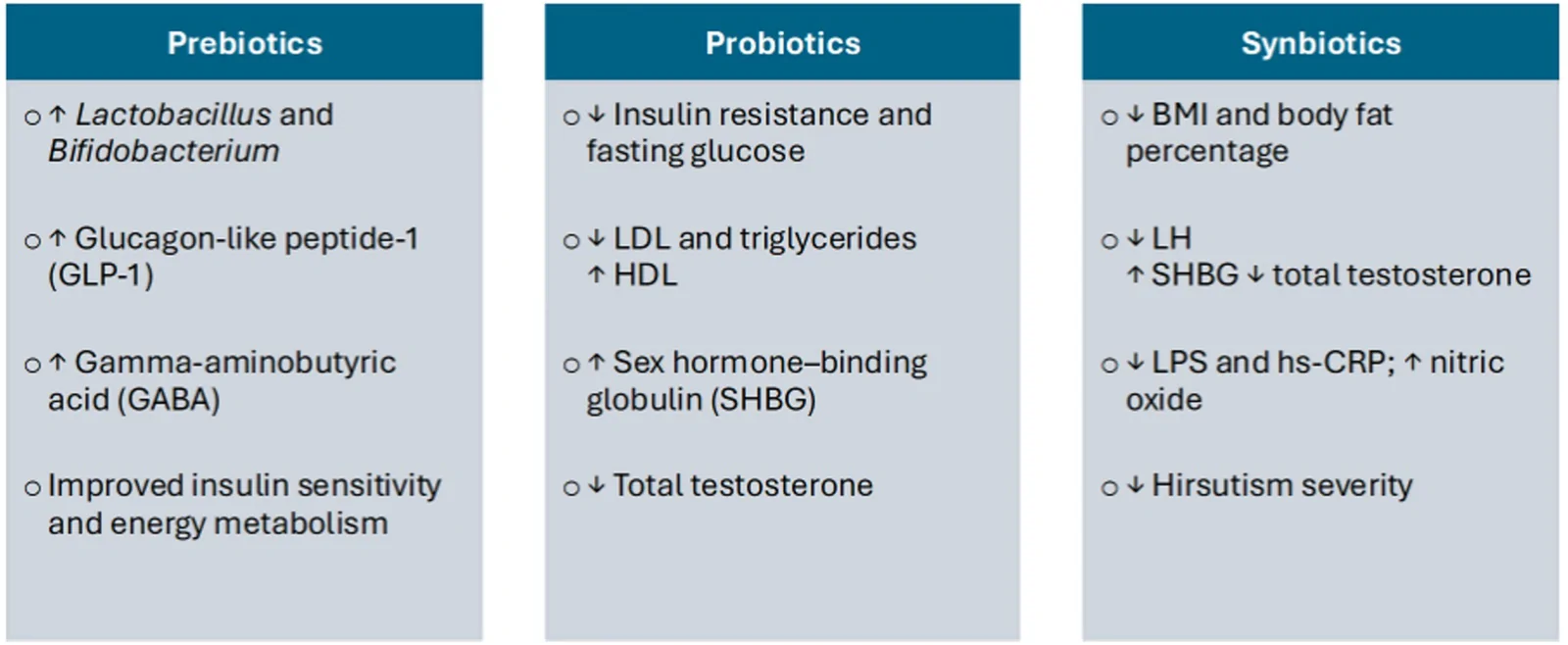
Summary of reported metabolic, hormonal, inflammatory and clinical effects of prebiotics, probiotics and synbiotics in women with PCOS. Arrows denote increases or decrease in measured outcomes. Figure created using Microsoft PowerPoint (Microsoft 365).

Among targeted microbiota-modulating strategies, probiotics, prebiotics and synbiotics each act through distinct but complementary mechanisms, as summarized in **Figure 3**. Probiotics are live beneficial microorganisms, most commonly Lactobacillus and Bifidobacterium species, that confer health benefits when consumed in adequate amounts. Prebiotics are non-digestible dietary components, such as inulin or fructooligosaccharides, that selectively stimulate the growth and activity of these beneficial microbes. Synbiotics combine probiotics and prebiotics in a single formulation, aiming to enhance microbial survival, colonization and functional activity within the host (13). Together, these interventions operate along a continuum of gut modulation, differing in whether they introduce beneficial bacteria directly, promote their endogenous growth, or synergistically support both processes.

### 6.1 Prebiotics

Although clinical data on isolated prebiotic supplementation in PCOS are limited, mechanistic evidence highlights their potential relevance. Most evidence regarding the effects of prebiotics on GLP-1 secretion and GABA production is derived from experimental studies and clinical investigations in healthy individuals or other metabolic conditions, rather than women with PCOS. Prebiotic intake has been shown to increase the abundance of *Bifidobacterium* spp. and stimulate colonic glucagon-like peptide-1 (GLP-1) secretion, a hormone involved in glucose homeostasis and insulin sensitivity. In addition, certain *Lactobacillus* and *Bifidobacterium* species promoted by prebiotics can synthesize gamma-aminobutyric acid (GABA), suggesting a potential mechanism through which the gut microbiota may influence energy metabolism and insulin secretion (44). These mechanisms align with the SCFA-mediated metabolic regulation discussed in Section 5.1. However, dedicated clinical studies are needed to determine whether these mechanisms translate into meaningful therapeutic benefits in women with PCOS.

### 6.2 Probiotics

Several trials of probiotic supplementation have reported potential benefits in improving metabolic and endocrine outcomes in women with PCOS. Across several clinical trials, probiotics reduced insulin resistance markers, improved fasting glucose, and favorably altered lipid profiles through reductions in low density lipoproteins (LDL) and triglycerides and increases in high density lipoproteins (HDL). Hormonal improvements were also notable, including increased sex hormone-binding globulin (SHBG) and decreased total testosterone (13).

One multi-strain probiotic formulation (*Lactobacillus acidophilus, Lactobacillus casei, Bifidobacterium bifidum*) was associated with increased SHBG and total antioxidant capacity while reducing testosterone, hirsutism scores, high-sensitivity C-reactive protein (hs-CRP), and malondialdehyde (MDA) levels (45). These findings are consistent with probiotic-mediated attenuation of LPS-driven inflammation and restoration of SCFA production, as outlined in Sections 5.1 and 5.2. Combining probiotics with selenium not only improved inflammatory and oxidative stress markers such as hs-CRP, MDA, and total antioxidant capacity (TAC), but also resulted in significant reductions in depression, anxiety, and stress scores (46).

Preclinical evidence also supports the biological plausibility of these findings: Lactobacillus supplementation in letrozole-induced PCOS rats restored estrous cycling, normalized ovarian morphology, reduced androgen biosynthesis and corrected dysbiosis (47).

### 6.3 Synbiotics

Synbiotics, combining probiotic and prebiotic components, may have broader metabolic, inflammatory, and hormonal regulatory potential, but their superiority over probiotics or prebiotics requires validation in direct comparative studies. Across several clinical studies involving overweight or obese women with PCOS, synbiotic supplementation was associated with improvements in insulin resistance, fasting glucose, lipid parameters, and hormonal disturbances, including increased SHBG and reduced testosterone (13, 48, 49). Although these findings are encouraging, direct comparisons with probiotics or prebiotics alone remain limited, precluding conclusions regarding comparative efficacy.

Additional studies have reported reductions in BMI, body fat percentage, LH, LPS, and LPS-binding protein following synbiotic supplementation (48). The breadth of these effects may reflect the combined capacity of synbiotics to restore SCFA production, reduce endotoxemia and modulate bile acid signaling, as discussed in Sections 5.1–5.3. Other trials also observed improvements in nitric oxide, hs-CRP, and hirsutism scores (49).

Synbiotics were also associated with improvements in patient-reported outcomes. A triple-blind clinical trial found that synbiotic supplementation improved several PCOS-specific quality-of-life domains including emotional wellbeing, body hair perception, weight concerns, and infertility-related distress, as well as overall physical functioning (50). These enhancements suggest that the physiological improvements observed with synbiotics translate into meaningful improvements in daily functioning and symptom burden.

**Figure 4:**
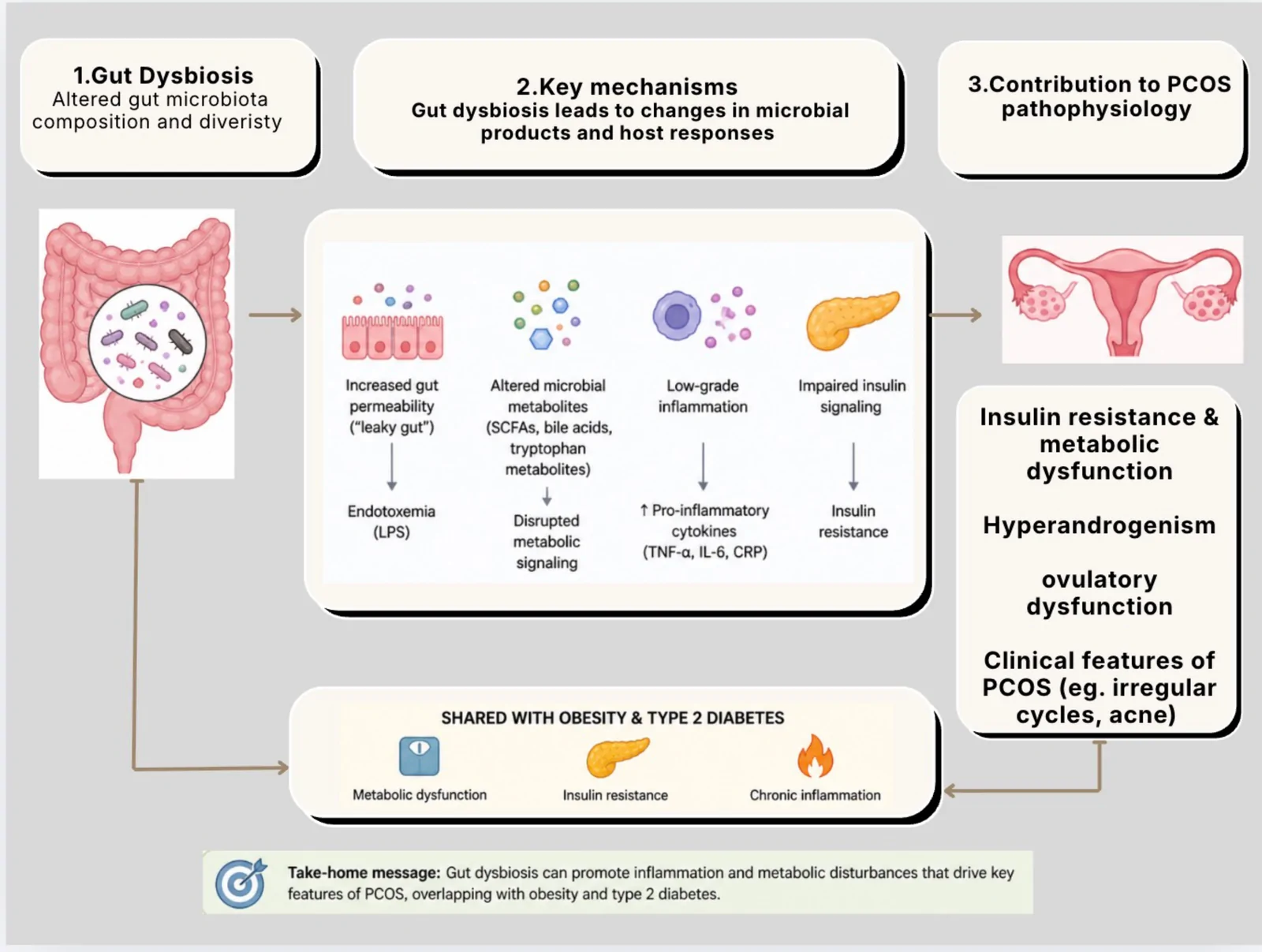
A simplified overview of the link between gut microbiome and PCOS. Figure created using Canva (Canva Pty Ltd).

Finally, when combined with functional foods such as pomegranate juice, synbiotics appeared to provide additional improvements by improving lipid profile, oxidative stress markers, systemic inflammation and blood pressure, reinforcing the potential synergy between microbiota-modulating supplements and antioxidant-rich dietary components (42). **Figure 4** illustrates the effects of microbiota-modulating therapies on metabolic and hormonal outcomes in PCOS.

### 6.4 Fecal microbiota transplantation

Fecal microbiota transplantation (FMT) represents a distinct and potentially more comprehensive microbiome-modulating strategy that warrants consideration in the context of PCOS. Unlike probiotics, which introduce select bacterial strains, FMT transfers the entire gut microbial ecosystem from a healthy donor, encompassing the bacteriome, virome, fungome, archaeome and parasitome, thereby offering a more holistic approach to restoring gut microbial homeostasis (51).

Preclinical evidence supports the therapeutic potential of FMT in PCOS. In the study by Guo et al. (47), FMT from healthy rats restored estrous cycles in 100% (8/8) of letrozole-induced PCOS rats, compared to 75% (6/8) with Lactobacillus transplantation alone. FMT also normalized androgen biosynthesis, improved ovarian morphology, and restored gut microbiota composition, suggesting that the transfer of a complete microbial community may confer advantages over single-strain probiotic interventions (47). A systematic review by Hanna et al. (14) identified FMT as a more promising but invasive option compared to probiotics, offering live bacteriotherapy as a potential therapeutic alternative for PCOS, though the authors emphasized the need for standardized protocols and larger-scale studies (14). Additionally, it has been hypothesized that combining FMT with maintenance agents such as curcumin may help sustain eubiosis and reduce the need for repeated transplantation, although this remains speculative and untested in clinical settings (51).

Despite these encouraging preclinical results, FMT remains at an early experimental stage in the context of PCOS. No human randomized controlled trials have yet evaluated FMT specifically for this condition (14, 52), and substantial practical barriers persist — including the absence of standardized donor screening and preparation protocols, potential infection risk, variable patient and clinician acceptance, and an evolving regulatory landscape. Unlike probiotics, prebiotics and synbiotics, which are supported by at least some clinical trial data in PCOS populations, FMT currently lacks any human efficacy or safety evidence in this setting. Moving forward, well-designed pilot studies in women with PCOS are needed to establish feasibility, preliminary efficacy and safety, alongside work to define optimal donor selection criteria, delivery methods and durability of microbiome engraftment.

### 6.5 Adverse effects and safety of probiotic, prebiotic, and synbiotic supplementation

The safety profile of microbiota-targeted interventions merits careful consideration alongside their therapeutic potential. Across the reviewed literature, probiotic, prebiotic and synbiotic supplementation was generally well tolerated. A meta-analysis of 17 RCTs encompassing 1,214 participants reported high adherence rates and no significant adverse effects alongside improvements in insulin resistance, lipid profiles and hormonal parameters (16). An umbrella review of 28 meta-analyses similarly identified no major safety concerns, with moderate-to-high certainty evidence supporting metabolic benefits (17), and an overview of systematic reviews corroborated these findings (18).

That said, the absence of reported adverse events should not be equated with confirmed long-term safety. Trial durations have typically been limited to 8–12 weeks with sample sizes under 100, which is insufficient to capture rare, cumulative or delayed effects (16, 18). Broader probiotic safety literature, outside the PCOS context, raises theoretical concerns including bacteremia risk in immunocompromised hosts, horizontal transfer of antibiotic resistance genes, gastrointestinal symptoms such as bloating and flatulence, and inconsistencies in commercial supplement quality and standardization (53).

Several population-specific considerations also remain unaddressed. Given that PCOS predominantly affects women of reproductive age, many of whom are actively seeking fertility, the safety of these supplements during pregnancy and the periconceptional period is a notable gap. Moreover, safety data generated with one bacterial strain cannot be assumed to apply to others, and potential interactions with medications commonly prescribed in PCOS, particularly metformin and oral contraceptives, have not been systematically evaluated. Addressing these gaps will require trials with longer follow-up, larger and more diverse cohorts, standardized adverse event reporting and dedicated assessments in pregnant or fertility-seeking populations.

## 7 Discussion

This narrative review highlights the increasingly recognized role of the gut microbiome in the pathophysiology and clinical expression of PCOS. The evidence reviewed here spans observational, preclinical and causal inference approaches, collectively suggesting that gut dysbiosis may function as both a consequence and a contributor to PCOS pathophysiology — though the strength of this evidence varies substantially across pathways. Recent bidirectional Mendelian randomization studies further support the possibility that specific microbial taxa may contribute to PCOS susceptibility while also indicating that disease-related metabolic disturbances may themselves influence gut microbiota composition, reinforcing the likelihood of bidirectional interactions. Accordingly, interpretation of the current evidence requires careful distinction between observational associations, mechanistic hypotheses, and causal findings.

### 7.1 Which mechanisms are most strongly supported?

Among the mechanistic pathways reviewed, the bile acid–FXR–IL-22 axis currently has the strongest evidence base in PCOS, supported by the study by Qi et al. (25), which combined human microbiome profiling with functional validation in gnotobiotic mice (25). The LPS–TLR4–NF-κB endotoxemia pathway is well established in obesity and type 2 diabetes mellitus (T2D) and is supported by strong associative evidence in PCOS, with elevated circulating LPS and LPS-binding protein correlating with hyperandrogenism and metabolic dysfunction (38, 39). Nevertheless, direct *in vivo* evidence demonstrating that endotoxemia induces ovarian androgen excess in women with PCOS remains lacking. The SCFA deficiency pathway, while mechanistically plausible, lacks direct PCOS-specific interventional validation (7, 28). In contrast, although the estrobolome has attracted increasing interest, direct evidence linking altered estrogen-metabolizing microbial communities to PCOS pathogenesis remains limited, and this pathway should currently be regarded as a promising but largely hypothetical mechanism requiring further validation (30, 40).

### 7.2 PCOS-specific versus shared mechanisms

An important question is whether gut dysbiosis in PCOS represents a disease-specific phenomenon or reflects the broader metabolic dysfunction shared with obesity and T2D. Current evidence suggests that both explanations likely contribute. The bile acid–IL-22 pathway appears relatively specific to PCOS, with *Bacteroides vulgatus*-mediated suppression of IL-22 contributing to ovarian dysfunction independently of obesity (25). In contrast, SCFA deficiency, metabolic endotoxemia, impaired gut barrier function, and chronic low-grade inflammation are common features across PCOS, obesity, and T2D, reflecting overlapping pathogenic mechanisms (31, 32, 35, 41).

The persistence of gut dysbiosis in lean women with PCOS further suggests that obesity alone cannot fully explain microbiome alterations. Instead, obesity-independent mechanisms involving bile acid signaling, immune-ovarian interactions, hormonal regulation, and potentially the estrobolome may contribute to disease pathogenesis in this subgroup, although direct evidence remains limited. Future studies should determine whether distinct microbial signatures characterize lean versus obese PCOS and whether microbiota-targeted interventions produce differential responses across these phenotypes.

Comparative analysis of gut microbiota across PCOS, obesity, and T2D demonstrates both shared and distinct microbial alterations. All three conditions are characterized by reduced SCFA-producing bacteria and increased markers of endotoxemia. However, certain microbial changes appear more disease-specific; for example, increased abundance of *B. vulgatus* has been reported more consistently in PCOS (25), whereas depletion of *Akkermansia muciniphila* is more strongly associated with obesity and T2D (31). Whether microbial therapies that improve insulin sensitivity in T2D provide equivalent benefits in PCOS remains uncertain, highlighting the need for disease-specific therapeutic approaches.

### 7.3 Critical appraisal of intervention evidence

Dietary interventions targeting the gut microbiome have emerged as promising adjunctive approaches for improving selected metabolic and inflammatory features of PCOS. Among the interventions reviewed, synbiotics have demonstrated the broadest range of reported improvements across metabolic, hormonal, inflammatory, and quality-of-life outcomes (15, 42, 48–50). Likewise, supplementation with *Lactobacillus* and *Bifidobacterium* strains has been associated with increases in sex hormone-binding globulin (SHBG) and reductions in total testosterone in several randomized trials (45, 46). These findings suggest potential endocrine benefits, although considerable uncertainty remains regarding the optimal strains, dosages, treatment duration, and patient populations most likely to benefit.

Importantly, the current clinical evidence base has several important limitations. Most randomized controlled trials enrolled relatively small numbers of participants, limiting statistical power and external validity. Intervention durations were generally short (8–12 weeks), precluding assessment of sustained clinical benefits or long-term safety. Considerable heterogeneity exists in probiotic strains, synbiotic formulations, dosages, and treatment protocols, making direct comparison across studies difficult. Clinical endpoints also varied substantially, and clinically meaningful reproductive outcomes, including ovulation rate, pregnancy rate, live birth rate, and validated assessments of hirsutism and acne,were inconsistently reported. Furthermore, many studies did not specify participants’ PCOS phenotypes, introducing additional clinical heterogeneity and limiting interpretation of treatment effects.

Although synbiotics appeared to demonstrate broader benefits than probiotics or prebiotics alone, this observation is derived from indirect comparisons across independent studies rather than direct head-to-head trials. Consequently, current evidence does not support conclusions regarding the superiority of one microbiota-targeted intervention over another.

Qualitatively, the certainty of evidence appears low to moderate for metabolic outcomes such as insulin resistance and lipid metabolism, whereas evidence supporting improvements in reproductive and other clinically meaningful outcomes remains low because of small sample sizes, methodological heterogeneity, indirectness, and limited follow-up.

Importantly, the available evidence also suggests that different microbiota-targeted interventions may influence distinct but overlapping biological pathways. Prebiotics primarily enhance the growth of endogenous SCFA-producing bacteria, thereby improving gut barrier integrity and reducing metabolic inflammation. Probiotics may attenuate endotoxemia, modulate inflammatory signaling, and influence endocrine function through alterations in microbial composition and metabolite production. Synbiotics may combine these complementary mechanisms by simultaneously introducing beneficial microorganisms while supporting their colonization and metabolic activity. However, direct evidence linking individual interventions to restoration of specific mechanistic pathways in women with PCOS remains limited, and most proposed mechanisms continue to be inferred from preclinical or non-PCOS studies rather than demonstrated in human clinical trials.

Fecal microbiota transplantation (FMT) should currently be regarded as an experimental strategy. Although preclinical studies demonstrate restoration of estrous cyclicity, normalization of androgen levels, and improvement of gut microbial composition in animal models of PCOS (47), as noted in Section 6.4, no randomized controlled trials have evaluated FMT in women with PCOS Together with challenges related to donor selection, infection risk, regulatory oversight, and long-term safety, this lack of clinical evidence precludes recommendation of FMT outside research settings.

### 7.4 Implications for clinical practice

At present, microbiota-targeted interventions should be viewed as promising adjunctive strategies rather than established components of routine PCOS management. Existing evidence suggests potential benefits for selected metabolic, inflammatory, and hormonal outcomes; however, it remains insufficient to recommend specific probiotic strains, synbiotic formulations, treatment durations, or patient selection criteria. Similarly, evidence supporting improvements in ovulation, pregnancy, live birth, hirsutism, acne, and other patient-centered clinical outcomes remains preliminary.

Broader dietary approaches, particularly Mediterranean and low-glycemic-index dietary patterns, appear to improve metabolic health partly through favorable modulation of gut microbial composition, increased dietary fiber intake, and enhanced production of beneficial microbial metabolites. Given their established cardiometabolic benefits beyond microbiome modulation, these dietary strategies currently represent the most practical and evidence-supported microbiota-oriented approaches for women with PCOS (43).

Overall, the available evidence supports an important role for the gut microbiome in PCOS pathophysiology while also emphasizing the complexity and heterogeneity of microbiome-host interactions. Although mechanistic insights and early clinical trials are encouraging, important gaps remain regarding causality, phenotype-specific responses, optimal therapeutic strategies, and long-term clinical efficacy. Continued integration of mechanistic studies, causal inference approaches, multi-omics analyses, and well-designed randomized clinical trials will be essential to determine whether microbiota-targeted therapies can ultimately become evidence-based components of personalized PCOS management.

### 7.5 Strengths and limitations

This review provides an integrated overview of the metabolic, endocrine, inflammatory, and microbiome-related mechanisms implicated in PCOS, while critically evaluating current microbiota-targeted interventions and incorporating recent advances in causal inference and multi-omics research. By discussing PCOS heterogeneity, evidence quality, and translational challenges, it offers a balanced perspective on the current state of the field.

However, as a narrative review, it is inherently subject to selection bias and does not include the systematic screening or quality assessment of a systematic review. The available evidence is also limited by heterogeneity in study design, participant characteristics, intervention protocols, and outcome measures, with many studies being cross-sectional and therefore unable to establish causality. Inconsistent control of confounding factors, including diet, medications, and antibiotic use, together with limited long-term safety data and potential publication bias, further constrain the interpretation and generalizability of current findings. The key mechanistic pathways, proposed relationships, and available intervention studies are summarized in **Tables 2**–**4**.

**Table 2:** Key mechanistic pathways linking gut dysbiosis to PCOS, obesity, and type 2 diabetes.

Mechanistic pathway	Key mediators	Effect on host	Relevance to PCOS	Shared with obesity/T2D	Evidence level in PCOS	References
SCFA deficiency	Acetate, propionate, butyrate; GPR41/GPR43	Impaired GLP-1/PYY secretion, reduced gut barrier integrity, increased inflammation	Worsened insulin resistance, impaired ovarian function	Yes, reduced SCFA-producing taxa in both conditions	Observational (cross-sectional microbiome studies)	(7, 28, 31, 41)
Endotoxemia (LPS-TLR4)	LPS, TLR4, NF-κB, JNK	Chronic low-grade inflammation, IRS-1 serine phosphorylation	Hyperandrogenism (LPS stimulates thecal androgen production), insulin resistance	Yes, central mechanism in obesity-associated inflammation and T2D	Associative (elevated LPS in PCOS cohorts); *in vitro* (LPS stimulates thecal androgens)	(31, 33, 34, 41)
Bile acid dysregulation	GDCA, TUDCA; FXR, TGR5	Altered FGF19, impaired GLP-1 secretion, reduced IL-22	Ovarian dysfunction via IL-22 deficiency, metabolic dysregulation	Yes, disrupted FXR/TGR5 signaling in obesity and T2D	Strong — human microbiome + gnotobiotic mouse validation (Qi et al., 2019); preclinical (Zhao et al., 2026)	(25, 36, 39)
Estrobolome dysfunction	β-glucuronidase, estrogen metabolism	Altered circulating estrogen levels	Anovulation, hormonal imbalance	Partially, estrogen metabolism affects adiposity	Hypothetical — no direct PCOS-specific evidence	(30, 40, 42)
Gut-brain axis	SCFAs, tryptophan metabolites, vagal signaling	Altered appetite regulation, neuroendocrine signaling	Dysregulated GnRH pulsatility, elevated LH/FSH ratio	Yes — appetite and energy homeostasis in obesity	Hypothetical — inferred from general neuroendocrine research	(30, 40)
Immune-inflammatory cascades	TNF-α, IL-6, IL-1β; macrophage pyroptosis	Systemic inflammation, impaired insulin signaling	Follicular arrest, metabolic syndrome features	Yes — adipose tissue inflammation in obesity, β-cell dysfunction in T2D	Associative (elevated cytokines in PCOS); *in vitro*/animal models	(19, 31, 34)

FGF19, fibroblast growth factor 19; FSH, follicle-stimulating hormone; FXR, farnesoid X receptor; GDCA, glycodeoxycholic acid; GLP-1, glucagon-like peptide-1; GnRH, gonadotropin-releasing hormone; GPR41/GPR43, G protein-coupled receptors 41 and 43; IL-1β, interleukin-1 beta; IL-6, interleukin-6; IL-22, interleukin-22; IRS-1, insulin receptor substrate-1; JNK, c-Jun N-terminal kinase; LH, luteinizing hormone; LPS, lipopolysaccharide; NF-κB, nuclear factor kappa B; PYY, peptide YY; SCFA, short-chain fatty acids; T2D, type 2 diabetes; TGR5, Takeda G protein-coupled receptor 5; TLR4, Toll-like receptor 4; TNF-α, tumor necrosis factor alpha; TUDCA, tauroursodeoxycholic acid.

**Table 3:** A summary of the proposed relationships between specific dysregulated pathways, targeted microbiota-modulating interventions and the phenotypic improvements observed or hypothesized in women with PCOS.

Dysregulated pathway	Targeted intervention	Proposed mechanism of action	Observed/Expected phenotypic improvement	Evidence level
SCFA deficiency → impaired GLP-1, gut barrier dysfunction	Prebiotics; fiber-rich diets (Mediterranean, low-GI)	↑ SCFA-producing bacteria → ↑ butyrate, propionate, acetate → ↑ GLP-1/PYY secretion, ↑ tight junction integrity	↓ Insulin resistance, ↓ intestinal permeability, improved glucose homeostasis	Mechanistic (general metabolic studies); limited PCOS-specific clinical data
LPS-mediated endotoxemia → NF-κB activation, chronic inflammation	Probiotics (Lactobacillus, Bifidobacterium); synbiotics	↓ Gram-negative bacteria, ↑ gut barrier integrity → ↓ LPS translocation → ↓ TLR4/NF-κB activation	↓ hs-CRP, ↓ LPS, ↓ inflammatory cytokines, ↓ insulin resistance	RCT evidence (small trials, 8–12 weeks)
Bile acid–FXR–IL-22 axis dysregulation	FMT (preclinical); synbiotics (theoretical)	Restoration of bile acid-metabolizing bacteria → ↑ TUDCA/GDCA → ↑ IL-22 via ILC3 → improved gut barrier and ovarian function	Restored estrous cycling, ↓ androgens, normalized ovarian morphology (animal models)	Strong preclinical; no human RCTs
Estrobolome dysfunction → altered estrogen metabolism	Probiotics (theoretical)	↑ β-glucuronidase-producing bacteria → ↑ estrogen reabsorption → improved estrogen signaling	Hypothetical: improved ovulatory function	Hypothetical — no PCOS-specific evidence
Immune-inflammatory cascades → follicular arrest	Probiotics + selenium; synbiotics	↓ Pro-inflammatory cytokines, ↓ oxidative stress → improved follicular environment	↓ hs-CRP, ↓ MDA, ↑ TAC, ↓ hirsutism scores	RCT evidence (small trials)
Gut-brain axis → dysregulated GnRH/LH	Synbiotics; dietary modification	Modulation of tryptophan metabolites, vagal signaling → improved neuroendocrine regulation	↓ Depression/anxiety scores, ↓ LH (indirect evidence)	Indirect/associative

The symbols “↑” and “↓” indicate an increase and decrease, respectively. The symbol “→” indicates a directional pathway, representing that one factor leads.

**Table 4:** Provides an overview of studies assessing microbiome-modulating interventions in PCOS.

Study	Design	Population	Intervention	Targeted pathway	Duration	Key outcomes	Refs
Karamali et al., 2018	RCT, double-blind, placebo-controlled	Women with PCOS (n=60)	Multi-strain probiotic (*L. acidophilus, L. casei, B. bifidum*)	LPS/inflammation, oxidative stress	12 weeks	↑ SHBG, ↑ TAC; ↓ testosterone, ↓ hirsutism, ↓ hs-CRP, ↓ MDA	(45)
Jamilian et al., 2018	RCT, double-blind, placebo-controlled	Women with PCOS (n=60)	Probiotic + selenium co-supplementation	Inflammation, oxidative stress, gut-brain axis	12 weeks	↓ Depression, anxiety, stress scores; ↓ hs-CRP, ↓ MDA; ↑ TAC	(46)
Nasri et al., 2018	RCT, double-blind, placebo-controlled	Women with PCOS (n=60)	Synbiotic supplementation	LPS/inflammation, hormonal regulation	12 weeks	↑ NO; ↓ hs-CRP, ↓ hirsutism; improved hormonal status	(49)
Chudzicka-Strugała et al., 2021	RCT, double-blind, placebo-controlled	Overweight/obese women with PCOS	Synbiotic + lifestyle modifications	SCFA production, LPS/endotoxemia	12 weeks	↓ BMI (8% vs 5% placebo); ↓ testosterone (32% vs 6% placebo)	(48)
Hariri et al., 2024	RCT, triple-blind, placebo-controlled	Women with PCOS	Synbiotic supplementation	Multiple (metabolic, inflammatory, psychological)	Variable	↑ QoL domains (emotional, body hair, weight, infertility)	(50)
Esmaeilinezhad et al., 2020	RCT, triple-blind, placebo-controlled	Women with PCOS	Synbiotic + pomegranate juice	Oxidative stress, inflammation	8 weeks	↓ Lipids, ↓ oxidative stress, ↓ inflammation, ↓ blood pressure	(42)
Guo et al., 2016 (animal)	Controlled animal study	Letrozole-induced PCOS rats	*Lactobacillus* transplant and FMT	Bile acid–IL-22 axis, global microbiome restoration	Variable	Restored estrous cycles (75% Lactobacillus, 100% FMT); ↓ androgens; normalized ovarian morphology	(47)
Chudzicka-Strugała et al., 2025	RCT, placebo-controlled	Overweight/obese women with PCOS	Synbiotic + lifestyle modifications	LPS/endotoxemia, SCFA production	6 months	↓ BMI, ↓ body fat %; ↓ LH, ↓ LPS, ↓ LPS-binding protein	(48)

Summary of clinical and preclinical studies evaluating the effects of probiotic, synbiotic and Fecal microbiota transplantation (FMT) interventions on metabolic, hormonal, and inflammatory outcomes in PCOS.

RCT, randomized controlled trial; PCOS, polycystic ovary syndrome; SHBG, sex hormone-binding globulin; TAC, total antioxidant capacity; hs-CRP, high-sensitivity C-reactive protein; MDA, malondialdehyde; NO, nitric oxide; BMI, body mass index; QoL, quality of life; LPS, lipopolysaccharide; FMT, fecal microbiota transplantation.

The symbols “↑” and “↓” indicate an increase and decrease, respectively.

### 7.6 Research gaps and future directions

Future research should prioritize phenotype-stratified and adequately powered clinical trials to determine whether microbiota-targeted interventions differ in efficacy across PCOS subgroups, including lean and obese phenotypes. Greater integration of multi-omics approaches, longitudinal cohort studies, and Mendelian randomization analyses is needed to strengthen causal inference and identify functional microbial pathways that may serve as therapeutic targets. Standardization of intervention protocols, clinical endpoints, and long-term safety monitoring will improve comparability across studies and facilitate clinical translation. Finally, further investigation into mechanism-based therapies, including targeted microbial modulation and fecal microbiota transplantation, is warranted before these approaches can be considered for routine management of PCOS.

## 8 Conclusions

Three principal findings emerge from this review. First, among the mechanistic pathways linking the gut microbiome to PCOS, the bile acid–FXR–IL-22 axis has the strongest PCOS-specific evidence, supported by both human microbiome profiling and functional validation in gnotobiotic models, whereas SCFA deficiency, endotoxemia and estrobolome dysfunction remain supported primarily by associative or extrapolated evidence. Second, microbiota-targeted interventions — particularly probiotics and synbiotics — show promising but preliminary benefits for metabolic and inflammatory outcomes, though the evidence base is limited by small sample sizes, short trial durations, heterogeneous protocols and a lack of clinically meaningful reproductive endpoints. Third, PCOS heterogeneity is a critical and underappreciated variable; differences in BMI, insulin resistance status and phenotype likely influence both the nature of gut dysbiosis and the response to microbiota-targeted therapies, yet most existing studies fail to stratify by these factors.

Moving forward, three priorities will be essential for clinical translation: (1) phenotype-stratified, adequately powered RCTs with standardized protocols and clinically meaningful endpoints including ovulation, pregnancy and live birth rates; (2) integrated multi-omics studies to identify functional microbial signatures that can serve as therapeutic targets or biomarkers of treatment response; and (3) mechanism-targeted intervention designs that match specific dysregulated pathways to appropriate microbiota-modulating strategies, enabling a precision medicine approach to gut microbiome-based PCOS management.
