# Supplementary material for: The gut microbiome in polycystic ovary syndrome: mechanistic pathways and therapeutic potential of microbiota-targeted interventions
Source: Front Endocrinol (Lausanne). 2026 Aug 21;17:1900535. doi: 10.3389/fendo.2026.1900535 (PMC13542650; doi:10.3389/fendo.2026.1900535)
Supplement: Supplementary file 1 [file Table1.docx]

Supplementary materials:

Table 1. Search strategy.

| **Database** | **Search String** |
| --- | --- |
| **PubMed** | ("Polycystic Ovary Syndrome"[Mesh] OR "PCOS" OR "Polycystic Ovary Syndrome") AND ("gut microbiome" OR "gut microbiota" OR "gut flora" OR "microbiome" OR "dysbiosis") AND ("dietary interventions" OR "diet" OR "nutrition" OR "prebiotics" OR "probiotics") AND ("symptoms" OR "clinical features") Filters: English, 2016–2026 |
| **Scopus** | (TITLE-ABS-KEY("polycystic ovary syndrome" OR "PCOS")) AND (TITLE-ABS-KEY("gut microbiome" OR "gut microbiota" OR "gut flora" OR "dysbiosis")) AND (TITLE-ABS-KEY("dietary intervention*" OR "diet" OR "nutrition" OR "prebiotic*" OR "probiotic*")) AND (TITLE-ABS-KEY("symptoms" OR "clinical features")) AND (PUBYEAR > 2015 AND PUBYEAR < 2027) AND (LIMIT-TO(LANGUAGE, "English")) |
| **Google Scholar** | "polycystic ovary syndrome" OR PCOS AND "gut microbiome" OR "gut microbiota" OR dysbiosis AND "diet" OR "dietary interventions" OR prebiotics OR probiotics AND symptoms (Custom range: 2016–2026, English) |
